# The evolving epidemiological landscape of atrial fibrillation: trends, challenges, and opportunities for improved patient care

**DOI:** 10.1093/europace/euaf026

**Published:** 2025-02-28

**Authors:** Marco Zuin, Matteo Bertini, Giuseppe Boriani

**Affiliations:** Cardiology Unit, Department of Translational Medicine University of Ferrara, Azienda Ospedaliero-Universitaria S.Anna, Via Aldo Moro 8, Ferrara 44124, Italy; Cardiology Unit, Department of Translational Medicine University of Ferrara, Azienda Ospedaliero-Universitaria S.Anna, Via Aldo Moro 8, Ferrara 44124, Italy; Cardiology Division, Department of Biomedical, Metabolic and Neural Sciences, University of Modena and Reggio Emilia, Policlinico di Modena, Modena 41124, Italy

**This editorial refers to ‘Global, regional, and national burden of atrial fibrillation and atrial flutter from 1990 to 2021: sex differences and global burden projections to 2046—a systematic analysis of the Global Burden of Disease Study 2021’, by S. Tan *et al*., https://doi.org/10.1093/europace/euaf027.**

Atrial fibrillation (AF) and atrial flutter (AFL) are prevalent cardiac arrhythmias that significantly contribute to morbidity and mortality worldwide.^1^ Recent epidemiological studies indicate a concerning upward trend in the global burden of AF/AFL.^2–4^ In this issue of *Europace*, Tan *et al*.^5^ present a comprehensive analysis of the growing epidemiological burden of these conditions over the past three decades, highlighting significant sex differences in prevalence and incidence, as well as projections for future trends. The findings from this study, derived from the Global Burden of Disease (GBD) programme, have profound implications for public health strategies aimed at reducing the burden of AF/AFL. Indeed, their results reveal that the epidemiological burden of AF/AFL is particularly pronounced in regions with lower socio-demographic index, where healthcare resources may be limited. Furthermore, while men generally exhibit higher incidence rates of AF/AFL, women are experiencing a faster increase in disease burden.^5^ This trend raises critical concerns about the unique challenges women face in accessing timely treatment and effective disease management. Moreover, Tan *et al*.^5^ identify hypertension, increased body mass index, and alcohol use as primary risk factors contributing to AF/AFL. Intriguingly, the epidemiological burden associated with BMI is more pronounced among women, suggesting an urgent need for targeted interventions within this demographic scenario.^5^

Overall, the investigation by Tan *et al*.^5^ provides a worrying projection for future years regarding the management of AF/AFL but also offers significant and timely potential solutions for implementing current healthcare strategies for patients with these arrhythmias.^5^ While primary prevention remains the best treatment strategy,^6^ there is an urgent need to drastically implement cardiovascular preventive measures, especially in low- and middle-income regions where healthcare resources are scarce.^7^ To reduce the impact of the epidemiological burden of AF/AFL on healthcare systems, intensification and widespread diffusion of public health initiatives promoting a healthy diet, regular physical activity, moderation of alcohol consumption, and smoking cessation must be the first step to further improve overall cardiovascular health and reduce the AF/AFL epidemiological burden.^8^

Moreover, understanding where the epidemiological burden of AF/AFL is likely to increase over the coming years will allow for prioritization of intervention in these areas, particularly as populations continue to age.^9^ Additionally, addressing sex-specific needs is fundamental; indeed, women often face unique barriers within healthcare systems that may influence their health outcomes. Furthermore, investigating biological differences—such as the impact of menopause on AF/AFL—as well as how sex differences affect treatment outcomes will be crucial for optimizing management strategies tailored specifically for female patients.^10,11^ Future studies should also assess whether simplifying risk stratification for stroke, as recommended by the latest European Society of Cardiology guidelines using the CHA2DS2-VA score, will effectively streamline decision-making for thromboprophylaxis and enhance the use of oral anticoagulation in AF patients deemed ‘truly low-risk’.^12–14^

Although the GBD study provided modelled estimates and not real data, Tan *et al*.’s study provides valuable insights into trends and projections regarding AF/AFL epidemiological burden, several areas warrant further investigation.^5^ Public health efforts must focus on education and screening to ensure equitable access to modern pharmacological and electrophysiological treatments.^5^ To this regard, a holistic approach to managing AF/AFL, considering comorbidities, cognitive function, and frailty, emerges as a viable strategy for developing comprehensive care plans tailored to individual patient needs.^12,15^ The increasing trend in AF/AFL cases signifies not only an urgent public health concern but also an opportunity for innovation in healthcare delivery (*Figure 1*). As we strive for better health outcomes for all patients affected by these conditions, it is essential to prioritize efforts that address both gender disparities and broader determinants of health influencing atrial fibrillation and flutter globally.

**Figure 1:**
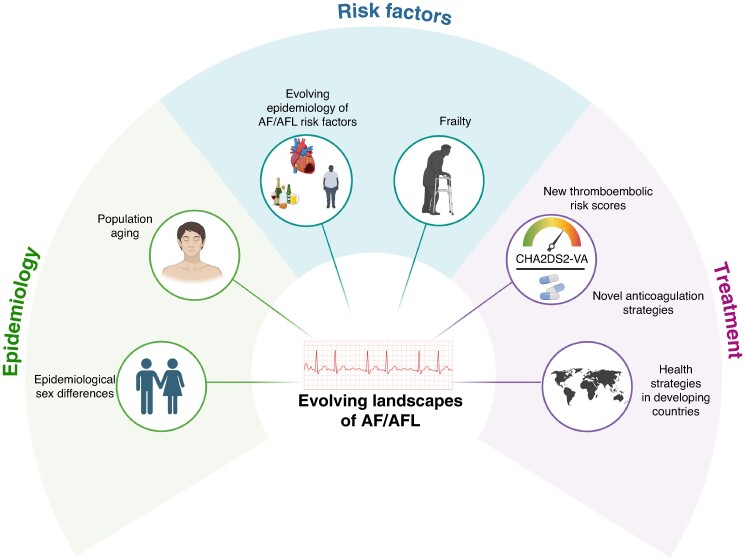
Evolving epidemiological landscape of atrial fibrillation and atrial flutter. AF, atrial fibrillation; AFL, atrial flutter.

In conclusion, Tan *et al*.’s study underscores the critical need for targeted interventions that consider sex differences in AF/AFL epidemiological burden, as well highlights the importance of proactive public health strategies aimed at reducing this growing global health challenge. By focusing on prevention, improving access to care, and tailoring management approaches to individual patient needs—particularly among women—we can significantly alleviate the epidemiological burden of AF and AFL in the years to come.

## Data Availability

No data has been used or generated for the present manuscript.
